# Impacts of cerium oxide nanoparticles on bacterial community in activated sludge

**DOI:** 10.1186/s13568-017-0365-6

**Published:** 2017-03-15

**Authors:** I. Kamika, M. Tekere

**Affiliations:** Department of Environmental Sciences, College of Agriculture and Environmental Science, University of South Africa (UNISA), PO Box 392, Florida, 1709 South Africa

**Keywords:** Bacteria, Activated sludge, Cerium, Nanoparticles, Nanotoxicity, EBPR

## Abstract

Rapidly developing industry raises concerns about the environmental impacts of nanoparticles, but the effects of inorganic nanoparticles on bacterial community in wastewater treatment remain unclear. The present research assessed the impact of cerium oxide nanoparticles (nCeO) on the microbiome of activated sludge system. The results showed that 18,330 over 28,201 reads generated from control samples were assigned to *Proteobacteria* while 5527 reads (19.6%), 3260 reads (11.567%), and 719 reads (2.55%) were assigned to unclassified_Bacteria, *Firmicutes* and *Actinobacteria*, respectively. When stressed with nCeO_2_ NPs, a decrease on reads was noted with 53, 48, 27.7 and 24% assigned to *Proteobacteria*. *Gammaproteobacteria* (80.57%) was found to be the most predominant *Proteobacteria*. The impact of nCeO_2_ NPs was also observed on pollutants removal as only 1.83 and 35.15% of phosphate and nitrate could be removed in the bioreactor stressed with 40 mg-nCeO_2_-NPs/L. This was confirmed by a drastic reduction of activities for enzymes catalysing denitrification (NaR and NiR) and degradation of polyphosphate (ADK and PPK). ADK appeared to be the most affected enzyme with activity decrease reaching over 90% when stressed with 10 mg-nCeO_2_/L. Furthermore, bacterial diversity was not significantly different whereas their species richness showed significant difference between control and treated samples. A large number of reads from control samples could not be classified down to the lower taxonomic level “genera” suggesting hitherto vast untapped microbial diversity. The denitrification related genera including *Trichococcus* and *Acinetobacter* were found to alternatively dominating treated samples highlighting those nCeO_2_ NPs could enhance the growth of some bacterial species while inhibiting those of others. Nevertheless, the study indicates that nCeO_2_ NPs in wastewater at very high concentrations may have some adverse effects on activated sludge process as they inhibit the removal of phosphate.

**Electronic supplementary material:**

The online version of this article (doi:10.1186/s13568-017-0365-6) contains supplementary material, which is available to authorized users.

## Introduction

In recent years, cerium oxide nanoparticles (CeO_2_ NPs) have been intensively studied owing to their wide applications and unique properties in UV absorbents and filters, gas sensors, catalytic wet oxidation, catalysts in the fuel cell technology, engine exhaust catalysts, photocatalytic oxidation of water, NO removal, electrolyzers, solid electrolytes and so on (Goharshadi et al. 2011). It has been reported that the ability of CeO_2_ NPs to express so many unique properties was mostly due to their ability to reversibly oxygenate and deoxygenate without disrupting the fluorite lattice-structure (Bumajdad et al. 2009). As rare metal, CeO_2_ NP chemical configuration with 4*f* orbitals buried inside the atom and shielded from the atom’s environment by the 4*d* and 5*p* electrons has also been seen as the main core of the expression of unique properties which is impossible with transition and main group metals (Hu et al. 2006; Bouzigues et al. 2011). According to Xu and Qu (2014), cerium atom can easily and drastically adjust its electronic configuration to best fit its immediate environment. Cerium oxide nanoparticles have also shown biological applications as they have been used as enzymes (e.g. superoxide oxidase, catalase, oxidase, etc.) and also as antioxidant or radical scavengers (Asati et al. 2009; Mandoli et al. 2010; Karakoti et al. 2010; Xu et al. 2013; Li et al. 2013). This unique nanoparticle has also been used to treat diseases such as oxidative stress-related diseases (e.g. neurodegenerative disorders), diabetes, retinal diseases, chronic inflammation and cancer (Maritim et al. 2003; Chen et al. 2006; Mariani et al. 2005; Lin et al. 2006; Federico et al. 2007; Hussain et al. 2012). Being a mature engineered nanoparticle with such unique properties and industrial applications, CeO_2_ NPs health and environmental concerns have mostly been overlooked. Recently, CeO_2_ NPs have been incriminated of being toxic to rats (Srinivas et al. 2011), freshwater alga (Taylor et al. 2015), human lung cells (Mittal and Pandey 2014), zebrafish (Arnold et al. 2013), and bacteria (Pelletier et al. 2010). Considering the above and through their lifecycles, CeO_2_ NPs represent a major concern as they are likely to enter natural water bodies channeled via wastewater treatment plants (WWTPs) (Aruoja et al. 2009). It is well-known that biological WWTPs consist of a series of biochemical processes, such as nitrification, denitrification, and phosphorus anaerobic release and aerobic or anoxic uptake engineered for the removal of nitrogen and phosphorus as well as other pollutants. The performance of these processes is directly related to the activities of some key microorganisms present in the activated sludge. However, it is unknown how nCeO_2_ NPs affect essential activities of these key microorganisms in activated sludge. Since activated sludge is the most widely used wastewater treatment option, the present study aimed at assessing the effect of nCeO_2_ NPs to activated sludge microorganisms.

## Materials and methods

### Bioreactors

Fresh activated sludge (1 L each) was collected from the Northern Wastewater Works, Johannesburg, chipped to the laboratory in a cooler box (4C) and used within 24 h. The collected activated sludge (100 mL) was then inoculated in a reactor containing 300 mL of culture media [d-glucose anhydrate (2.5 g/L), MgSO_4_·7H_2_O (0.5 g/L) and KNO_3_ (0.18 g/L) in distilled water] and treated with different concentration of CeO_2_ NPs (10, 20, 30 and 40 mg/L). In order to assess the impact of cerium oxide nanoparticles on the microbial community of wastewater treatment plants, the non-treated mixed liquor which contained the mixed liquor medium without nCeO_2_ NP was used as control. Experiments were run at 28 ± 2 °C on a checking incubator at 120 rpm for 5 days under aerobic condition. Aliquots were then taken at the final incubation day and analysis for microbial community. The aliquot samples were also used to determine the chemical oxygen demand (COD), nitrate and phosphate, pH, dissolved oxygen (DO) and electrical conductivity (EC). To test for NO_3_^−1^, the sodium salicylate method was used as reported by Monteiro et al. (2003). Briefly, 50 mL of samples was pipetted into milliliter beaker, and mixed with 1 mL of the salicylate solution. The mixture was dried out in an oven at 105 °C to allow the formation of NO_2_^+1^ from NO_3_^−1^. Then, 1 mL of sulfuric acid (17.4 M) was added and allowed to cool for 10 min and 7 mL of the solution containing sodium hydroxide (5 M) and sodium potassium tartrate (149 g/L) were later added. The solution was later made up with water and analysed in a spectrophotometer (Monteiro et al. 2003). For PO_4_^−3^, the method 424*f* standard method as reported by APHA (2001) was used. The method 424*f* uses ammonium molybdate and potassium antimonyl tartrate in order to react in an acidic medium with orthophosphate to form a heteropoly acid (phosphomolybdic acid) that is reduced to intensely coloured molybdenum blue by ascorbic acid. The closed reflux method was also used to measure COD concentration (APHA 2001), whereas pH, DO, electrical conductivity (EC) and temperature were measured using specific probes (HACH, Germany). All experiment was done in triplicates.

### DNA extraction, amplification and sequencing of bacterial 16S rRNA genes

In order to extract the genetic material (DNA) representing the microbial communities of each bioreactor, an aliquot (100 mL) of nCeO_2_-free and treated mixed liquor from day 5 samples was centrifuged at 10,000×*g* for 5 min at 4 °C and the collected cells cleaned twice using sterile phosphate buffer solution (1×). The collected cell pellets were re-suspended in 1× TE buffer (pH 8.0), homogenously mixed and DNA was extracted using the ZR Fungal/Bacterial DNA Kit™ (Zymo Research, Pretoria, South Africa) according to the procedures provided by the manufacturer. The integrity and purity of extracted DNA was further assessed on the 1.0% agarose gel and measured using a Nanodrop spectrophotometer (Nanodrop 2000, Thermo Scientific, Japan).

### Amplification and sequencing of bacterial 16S rRNA genes

Prior of sequencing, the extracted DNA was amplified in triplicate and the V3 and V4 regions of the 16S rRNA gene were targeted by using the universal primers pairs (341F and 785R) and pooled together in order to better sample rare organisms, and avoid PCR biases (Klindworth et al. 2013; Sekar et al. 2014). Each 50 μL PCR reaction system contained 25 µL of 2X Dream Taq green Master Mix (DNA polymerase, dNTPs and 4 mM MgCl2), 22 µL of sterile Nuclease-free water, 1 µL of forward primer (0.2 µM) and 1 µL of reverse primer (0.2 µM), and 1 µL of DNA (50–100 ng/µL). In order to control nuclease contamination, negative control was included at every reaction. The following PCR reaction was performed: an initial denaturation step at 94 °C for 5 min, followed by 30 cycles of denaturation at 94 °C for 1 min, annealing at 55 °C for 30 s and extension at 72 °C for 1 min 30 s, and a final extension at 72 °C for 10 min, followed by cooling to 4 °C. The PCR products were loaded in 1% (m/v) agarose gel (Merck, SA) stained with 5% of 10 mg/mL ethidium bromide (Merck, SA) and visualized under ultra violet Trans illuminatior (InGenius Bio Imaging System, Syngene, Cambridge, UK). The correct PCR amplicons of bacteria were pooled together for the respective samples at approximately equimolar concentrations and submitted to Inqaba Biotechnology Industries, South Africa for sequencing on an Illumina MiSeq.

### Enzyme essay

In order to investigate the impact of nCeO_2_ on functional microbial population in the bioreactor, enzymes catalysing the degradation of polyphosphate such as adenylate kinase (ADK) and polyphosphate kinase (PPK) as well as those involved in the denitrification process namely nitrate reductase (NaR) and nitrite reductase (NiR)were assessed. Prior to assess enzymatic activities, activated sludge aliquots was taken and cleaned three times with 1.5 M NaCl buffer 5 M NaCl buffer consisted with 0.01 M EDTA and 1 mM NaF (pH 7.4). Cell structure of activated sludge were later broken down by resuspending pellets and sonicating for 5 min at 20 kHz and 4 °C, and centrifuged for 10 min at 12,000 rpm as reported by Chen et al. (2012). ADK was determined by mixing 0.16 mL of cell extract per mL with 7 mM MgCl_2_, 90 mM Tris hydrochloride (Tris–HCl, pH 7.0), 200 mM d-glucose, 0.6 mM NADP (Sigma), 3.4 U of hexokinase (HK, Wako Chemical, Osaka, Japan), and 1.7 U of glucose 6-phosphate dehydrogenase (G6P-DH, Wako Chemical, Osaka, Japan) per mL. Adenosyne diphosphate (1 mM ADP) was later added to the mixture in order to start the enzymatic reaction and the production of NADPH2 was measured at 340 nm by microplate reader (BioTek, USA). For PPK activity, the polyphosphate utilization approach was used and the reaction was carried out at 30 °C after mixing 150 µL crude extracts with 100 mM Tris–HCl buffer (pH 7.4), 8 mM MgCl_2_, 200 mM d-glucose, 0.5 mM NADP, 150 µg of sigma Type 45 poly-P, 1 unit of HK and 1 U of G6P-DH (Chen et al. 2012). The enzymatic activity of PPK and ADK were defined as the production of _µmol NADPH/(min mg protein). As for denitrification process enzymes such as NaR and NiR enzyme, their enzymatic activities were assayed according to Kenji et al. (1981). One unit of enzyme activity for NaR and NiR was defined as the production of 1 μmol/(min mg protein).

### Assessment of nCeO_2_-NPs impact on microbial morphology

To further determine the impact of nCeO_2_-NPs on the microbial population, a scanning electron microscopy (SEM) was used. After 5 days of incubation, nCeO_2_-NPs treated and not treated samples were centrifuged (10 mL) at 7000×*g* at 4 °C for 10 min. Microbial pellets were later washed five times using 0.1 M phosphate buffered saline (1× PBS) and fixed for 24 h in 2% glutaraldehyde (prepared in 1× PBS). Pellets were further dehydrated through a series of ethanol starting from 60% to absolute, and for each series samples were held for 30 min. Samples were placed on a brass stub, sputter-coated with gold and examined by SEM.

### Statistical data processing

Prior to be used, artificial replicate reads and low quality reads were removed from the dataset using Mothur pipeline (Schloss et al. 2009). Good quality reads were further pre-screened for ribosomal identity (at approximately 70% identity) using Qiime-uclust and chimeras removed through UCHIME according to de novo method (Edgar et al. 2011). All rRNA non-chimeric reads were later been analyzed at a confidence threshold of 97% for taxonomic classification using RDP pyrosequencing pipeline. In addition, reads with similarity more than 97% were clustered within the same operational taxonomic unit (OTU) and rarefaction curves were also determined (Wang et al. 2007; Cole et al. 2014). The diversity index Shanon and richness estimator Chao1 were also performed to estimate the microbial diversity and richness from each water samples. The relative abundance (%) of individual taxa within each community was calculated by comparing the number of sequences assigned to a specific taxon against the number of total sequences obtained for that sample. The similarity and dissimilarity in bacterial community structure within both wastewater treatment plants were analyzed using Jaccard index (Cole et al. 2014). Generated data was later made publicly available at the DDBJ Sequence Read Archive (DRA) under the accession number PSUB005615.

## Results

### Community species richness and diversity indices

The present study generated approximately 28,201 reads from the control samples but when stressed with an increase nCeO_2_ concentration, samples showed an approximately 28.6% decrease (20,135 reads) to a 57.1% decrease (12,082 reads) in the samples treated with 10 mg/L-CeO_2_ and 40 mg/L-CeO_2_, respectively. Similar observation was noted with the operational taxonomic units (OTUs) as a total of 27,967 OTUs was generated from the control samples while the sample with highest nCeO_2_ NP revealed a total of 6433 OTUs. The impact of nCeO_2_ NPs on the microbial complexity and abundance in the samples was also revealed by using the Shannon–Weaver index and Chao1 richness estimator at 3% cutoff (Table 1). The diversity index (Shannon) revealed a fluctuation in diversity as Shannon values for each samples were not inversely proportional to the increase of nCeO_2_ NP in the reactors as sample containing 40 mg/L-nCeO_2_ had high diversity index (8.178) while those with 30 mg/L-nCeO_2_ NPs was the lowest (7.689). Besides the fact that control samples had the highest diversity index (10.267), no significant difference (p > 0.05) between treated samples in terms of diversity index was observed and this revealed that nCeO_2_ NPs impacted more on the microbial abundance than on the diversity. The evenness highlighting the complexity of individual microbial population within samples also revealed that no statistical difference between samples in terms of microbial complexity as the values ranged from 0.885 to 0.999. A species richness test conducted using Chao1 richness estimator showed a drastic decrease of species richness of approximately 97.23–98.48% when comparing the control samples to nCeO_2_ NP treated samples.

**Table 1:** Diversity indices of samples treated with nCeO_2_ NPs during 5 days of incubations

Sample ID	N	OTU	Chao1	Shannon index	Evenness index
Control	28,201	27,967	2,310,921.517	10.267	0.999
S_A (10 mg/L)	20,135	9805	63,911.937	8.135	0.885
S_B (20 mg/L)	14,632	7226	40,791.791	7.929	0.892
S_C (30 mg/L)	14,292	7193	35,100.622	7.689	0.877
S_D (40 mg/L)	12,082	6433	50,783.275	8.178	0.921

**Fig. 1:**
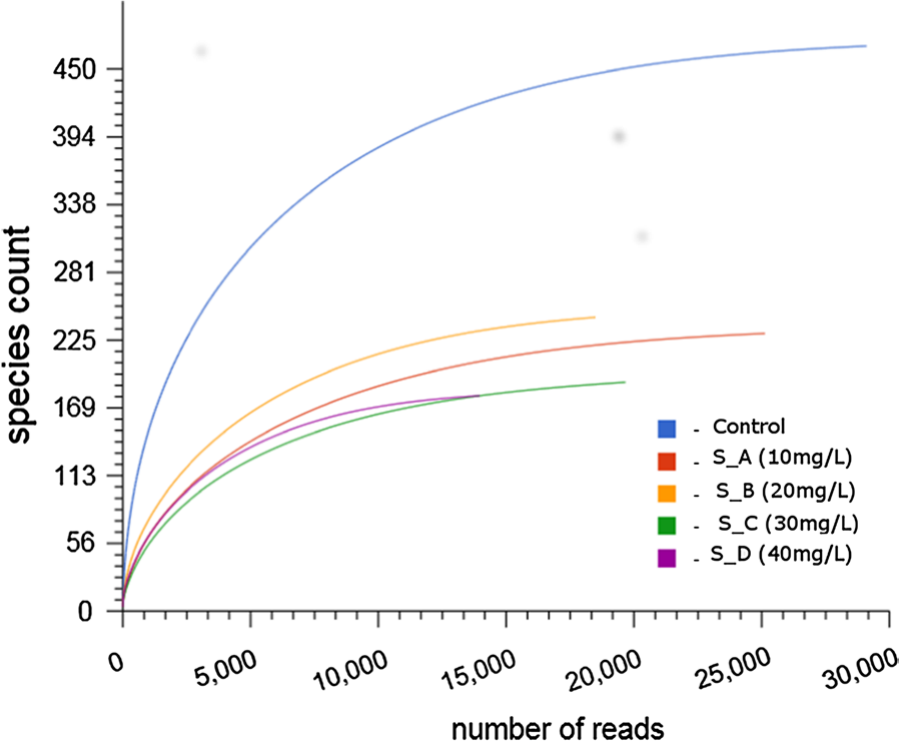
Rarefaction curves showing the dissimilarity levels among reactors treated and not treated with nCeO_2_ NPs

An additional confirmatory test on species richness conducted using rarefaction analysis also revealed a difference in the number of reads and OTUs between samples and control highlighting a high dissimilarity in bacterial diversity with control having more OTUs and reads than the treated samples. When comparing treated samples among them, no significant difference was noted (Fig. 1). However, the absence of plateau on the bacterial samples indicated that sequencing depth was still not enough to cover the entire bacterial diversity and a large fraction of the different species remains to be discovered. A pairwise community similarity between samples was assessed based on the absence and presence of each OTU using a Jaccard index (Additional file 1: Table S1). The Jaccard index exhibited a moderate or no similarity between all bacterial samples ranging with values from 0.479 to 0.999 with the S_A (10 mg/L) and S_C (30 mg/L) bacterial community showing the most similarity (0.479) as compared to others.

### Change of bacterial community in activated sludge over nCeO_2_ NP effects

**Fig. 2:**
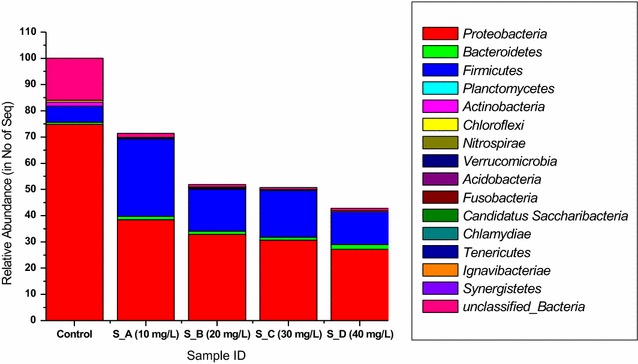
Taxonomic distribution of the most abundant bacterial phyla in both nCeO_2_ NPs-treated and not treated (control) samples. Analysis of 16S rRNA gene sequences was done in comparison with the RDP II database

**Fig. 3:**
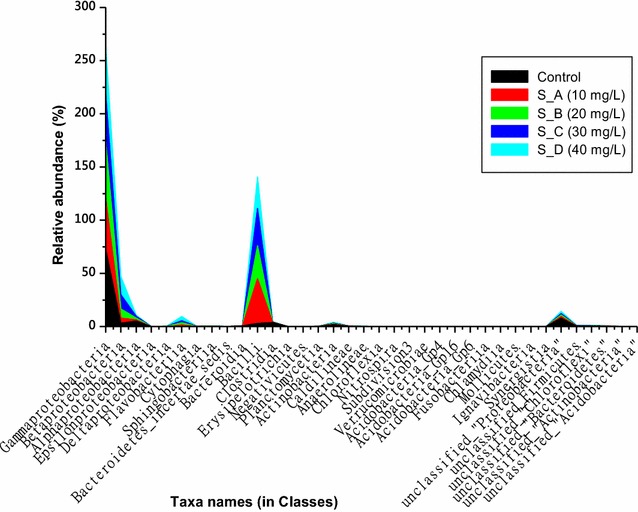
Relative abundance (%) of bacterial classes in nCeO_2_ NPs-treated and not treatment samples

In the present study, *Proteobacteria* has been noted as the most predominant phylum in our samples with an average number of reads of 18,330 out of 28,201 assigned to it in the control samples. Moreover, *Proteobacteria* dominated by *Gammaproteobacteria* (80.57% of the all population), *Alphaproteobacteria* (5.19%) and *Betaproteobacteria* (3.19%) was followed by unclassified bacteria (19.6%), *Firmicutes* (11.567%), *Actinobacteria* (2.55%) and other additional 11 phyla occupying only 1.5% of the all populations (Figs. 2, 3; Additional file 1: Table S2). The control showed an overall 15 phyla, 36 classes, 54 orders, 107 families and 240 genera. Furthermore, number of reads assigned for *Proteobacteria* appeared to decrease in the nCeO_2_ NP-treated samples as the concentration of test NPs increases. However, *Proteobacteria* was still noted to be the predominant phylum in the presence of 10 mg-nCeO_2_/L (53%) and 20 mg-nCeO_2_/L (48%). Unlike in control samples, in the nCeO_2_ NPs-treated samples, *Firmicutes* was the second most predominant phylum compared to unclassified bacteria in the control. This situation revealed that in our reactors nCeO_2_ NPs could promote the growth of some type of microorganisms while slowing the growth of others. Moreover, *Firmicutes* phylum was dominated by classes of *Bacilli* (29.49–41.86%) followed by *Clostridia* or unclassified *Firmicutes* (Fig. 3).

Even though the bacterial community appeared to be more diverse as the sequences were classified into lower taxonomic levels, their relative abundances were affected (Additional file 1: Tables S2–S5). Up to the order level, control samples (approximately 21,521 reads) revealed high abundance than the treated samples (19,303, 14,023, 13,840 and 11,501 reads from S_A, S_B, S_C and S_D, respectively). However, the control samples showed more unclassified sequences as compared to the treated samples leading to lower abundance at the family and genus level. When considering each sample individually, and the bacterial proportion in the lower taxonomic level “genera”, control samples were populated by 239 genera with unclassified_*Comamonadaceae* (26.61%), unclassified_*Moraxellaceae* (8.93%), unclassified_*Pseudomonadaceae* (7.08%), *Novispirillum* (5.88%), *Fusibacter* (4.88%), unclassified_*Enterobacteriaceae* (4.48), unclassified_*Xanthomonadaceae* (3.86%), *Shewanella* (3.05%), *Proteocatella* (2.93%), unclassified_*Carnobacteriaceae* (2.9%), *Acinetobacter* (2.84%), *Proteiniclasticum* (2.76%), *Trichococcus* (2.28%) and the remaining occupying a total of 21.51% (with < 2% each). Contrary in the nCeO_2_ NP-treated samples, unclassified genera appeared to be affect as their relative abundance drastically reduced to a range of 10 and 0%, while other classified genera showed their abundances increase (Additional file 1: Table S5). At the presence of 10 mg-nCeO_2_ NP/L, 123 genera were observed with *Trichococcus* (38.25%) was the most dominant genus followed by *Acinetobacter* (32.29%), unclassified_*Pseudomonadaceae* (8.9%), *Pseudoxanthomonas* (3.09%) and unclassified_*Enterobacteriaceae* (2.44%). In the samples treated with 20 mg-nCeO_2_ NPs/L, 115 genera were found with Acinetobacter (35.87%), *Trichococcus* (28.28%), unclassified_*Moraxellaceae* (6.9%), unclassified_*Comamonadaceae* (4.04%), *Aerococcus* (3.49%), *Shewanella* (2.72%), *Comamonas* (2.66%), unclassified_*Carnobacteriaceae* (2.1%) and the remaining with abundance <2% each. In the sample treated with 30 mg-nCeO_2_ NPs/L, the fluctuation was also noted as 108 genera was generated with *Trichococcus* (36.35%), *Acinetobacter* (33.85%), *Comamonas* (5.26%), unclassified_*Comamonadaceae* (5%), *Pseudoxanthomonas* (4.5%) and unclassified_*Moraxellaceae* (4.08%) as the most abundant genera in the samples. Similar to other treated samples, a total of 99 genera were observed in the 40 mg-nCeO_2_ NP/L treated samples with *Acinetobacter* (29.68%), *Trichococcus* (28.73%), unclassified_*Comamonadaceae* (10.1%), *Pseudoxanthomonas* (5.19%), *Comamonas* (4.74%), unclassified_*Moraxellaceae* (4.25%), *Aerococcus* (2.61%), *Cloacibacterium* (2.35%), and unclassified_*Pseudomonadaceae* (2.15%) as the most predominant genera. Despites the observed alternance, the treated samples have the same most two dominant genera as compared to the control samples. The study revealed that nCeO_2_ NPs affected the diversity of the microbial population while enhancing the growth of particular microbial species.

### Change of enzymatic activities in activated sludge stressed with nCeO_2_ NP

**Fig. 4:**
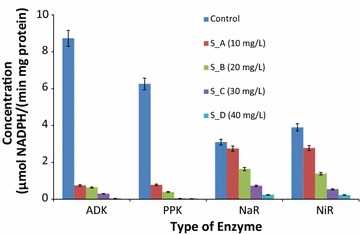
Effects of nCeO-NPs on activities of key enzymes associated with denitrification and polyphosphate degradation in EBPR. The unit is µmol NADPH/(min mg protein)

Figure 4 illustrates the impact of nCeO_2_-NP on the enzymatic activities in the activated sludge. It was observed that enzymatic activities of enzymes catalyzing the denitrification of nitrate (NaR and NiR) were less affected by nCeO_2_-NP than those catalyzing the degradation of polyphosphate (ADK and PPK). The statistical significant difference (p > 0.05) between activities of NaR-NiR and ADK-PPKwas noted. Despite the significant effect of nCeO_2_-NPs to ADK-PPK than to NaR–NiR, all enzymatic activities appeared to decrease over the increase of nCeO_2_-NPs in the media. Activities of ADK was the most affected with a decrease of 91.41–99.54% when compared to the control, while NaR showed the lowest decrease on activities ranging from 11.29 to 92.26%.

### Effects of nCeO_2_-NPs on microbial structure

**Fig. 5:**
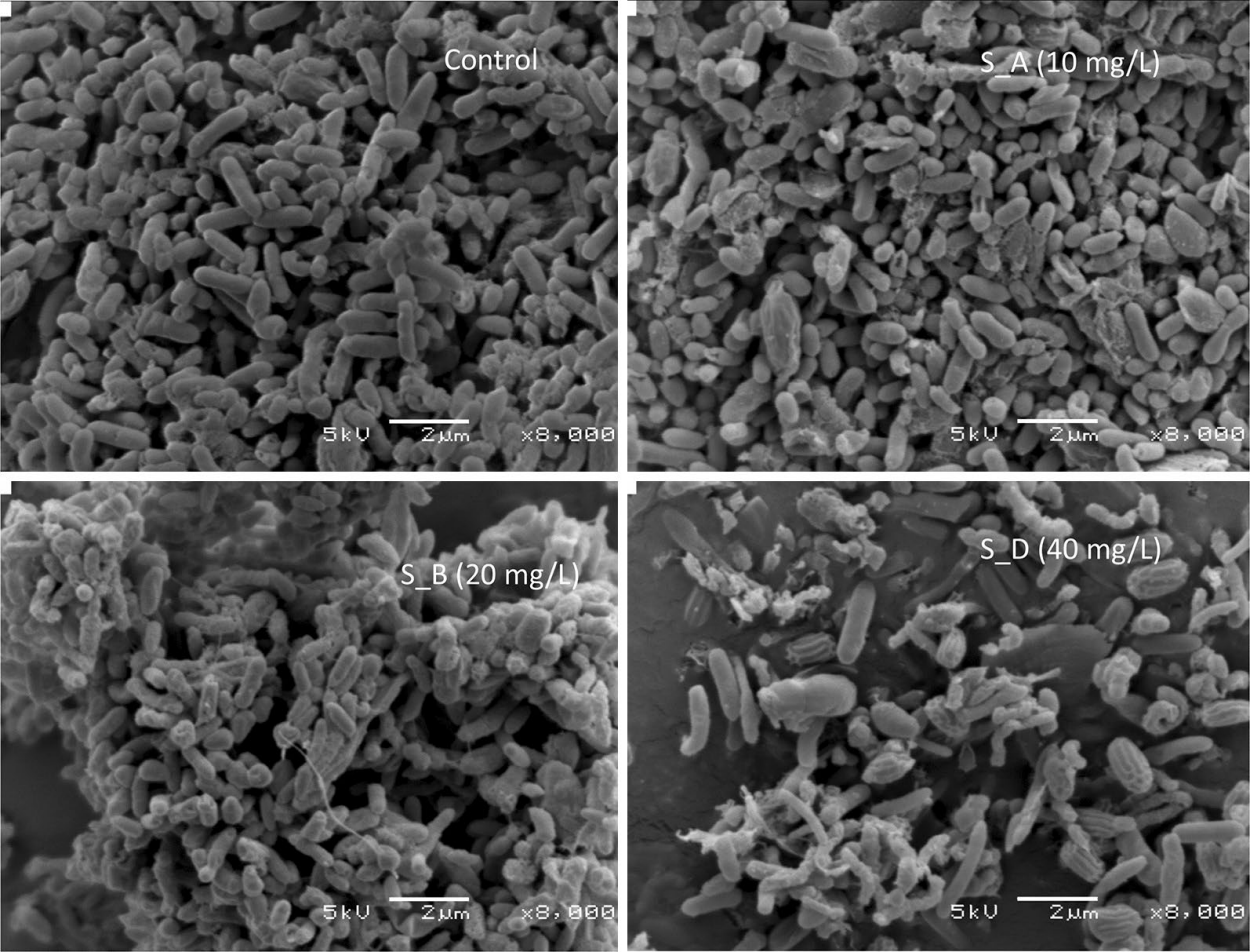
Scanning electron micrograph of microbial biomass in activated sludge stressed with cerium oxide nanoparticles (nCeO_2_-NPs)

The representative SEM images of the microbial biomass stressed for 5 days to nCeO_2_-NPs compared to the control (nCeO_2_-NPs free-sample) are shown in Fig. 5. The integrity of bacterial cell structure appeared to be disrupted leading to the agglomeration and lyse of microbial cells when compared to the microbial cells from the control sample. Control sample showed a high microbial biomass compared to nCeO_2_-treated samples with a decrease on microbial biomass as the concentration of nCeO_2_-NPs increases. SEM images further showed a heterogeneous microbial morphology in both nCeO_2_-NPs-treated and nCeO_2_-NPs-free samples. Rod-shaped microorganisms were noted to be predominant to the microbial community followed by cocci-shaped microorganisms.

### Variation of physicochemical parameters

Table 2 illustrates changes physicochemical parameters in the media under nCeO_2_ NP effects. It was noted that microbial community’s abilities in removing nitrate, phosphate and COD decreased as the nCeO_2_ NP increased in the media. Carbon oxygen demand was the parameters with the highest removal at 89.74% (initial concentration: 593.5 mg/L) followed by nitrate (75%) and phosphate (13.81%). Microbial community also showed uptake of dissolved oxygen at up to 65.09% from the culture media containing 6.2 mg-DO/L. However, the uptake was hampered as nCeO_2_ NP concentration was increasing. The present study revealed that there was not significant variation of electric conductivity and pH with the percentage variation ranging from 643 to 717 µS/cm and 7.21–7.18 pH unit, respectively during the experimental period.

**Table 2:** physicochemical changes in the culture media at the end of incubation (5 days)

	Initial Conc.	Control (%)	S_A (%)	S_B (%)	S_C (%)	S_D (%)
Phosphate (mg/L)	7.35	89.2	13.81	6.74	2.93	1.83
Nitrate (mg/L)	31.23	99.6	75	53.6	47.32	35.15
COD (mg/L)	593.5	95.98	89.74	71.21	54.18	39.45
EC (µS/cm)	717	53.64	10.32	6.93	4.72	2.81
DO (mg/L)	6.2	98.87	65.09	44.96	29.9	20.43
pH	7.21	12.9	0.21	0.38	0.28	0.26

## Discussion

For many decades now, activated sludge under the Enhanced biological phosphorus removal (EBPR) has widely been used as means for the treatment of wastewater (Seviour et al. 2003; Oehmen et al. 2007). Even though many EBPR wastewater treatment plant (WWTP) is considered as a cost-effective and eco-friendly process for the removal of phosphorus and other pollutants, this process is also prone to instability and unreliability which is often attributed to competition between detrimental and beneficial microbes in the plants (Sidat et al. 1997; Kamika et al. 2014). Regardless of the fact that other factors such as environmental as well as anthropogenic have been investigated for their role in the deterioration of EBPR, very few studies have been carried out on the impact of nanoparticles on the activated sludge microbial community (Khan et al. 2002; Thomsen et al. 2007: Zhang et al. 2012). To our knowledge, the impact of nCeO_2_ NPs on activated sludge microbial community as well as on the bacteria is still unknown. In the study, the impact of nCeO_2_ NP on the bacterial community structure and species richness using Illimina sequencing of 16S rRNA gene in order to understand the influences of NPs on the useful bacterial community in an activated sludge system. The present study revealed that out of the 28,201 reads generated from the control samples, 18,330 reads (64.77%) were assigned to *Proteobacteria* phylum while 5527 reads (19.6%), 3260 reads (11.56%), and 719 reads (2.55%) were assigned to unclassified_Bacteria, *Firmicutes* and *Actinobacteria*, respectively (Fig. 1). In general, a decrease on microbial abundance was noted in samples treated with nCeO_2_ NPs with 10,856 reads (38.49%), 9256 reads (32.82%), and 7671 reads (27.2%) assigned to *Proteobacteria* phylum in samples treated with 10, 20, 30 and 40 mg/L, respectively. Similarly to the present study, common phyla *Proteobacteria* and *Actinobacteria* have been reported in the activated sludge (EBPR) as they have involved in several mechanism such as phosphorus and nitrate removal from the waste (Liu et al. 2005; Sanz and Kochling 2007; Kamika et al. 2014). According to Kamika et al. (2014), classes belong to the *Proteobacteria* phylum such as *Gammaproteobacteria* (80.57% of the all population), *Alphaproteobacteria* (5.19%) and *Betaproteobacteria* (3.19%) have been reported as functional bacteria for EBPR. The present study also agreed with Chen et al. (2014) who reported that the addition of NPs such as ZnO-NP and Ag-NP have a remarkable impact to the functional bacterial community in activated sludge. To further investigate the impacts of nCeO_2_ NPs on the bacterial community/diversity, it was revealed that 18 phyla were generated from the control samples whereas in the nCeO_2_ NPs-treatment samples over 11 phyla, 13 phyla, 10 phyla and 10 phyla, in S_A (10 mg/L), S_B (20 mg/L), S_C (30 mg/L) and S_D (40 mg/L) samples, respectively. This was also confirmed as the diversity index (Shannon) and Chao1 richness estimator revealed a significant different (p < 0.05) between treated samples and the control samples. Unlike the control samples, no significant difference (p > 0.05) was noted within treated samples. A further confirmation was noted as the species richness test indicated a drastic decrease of approximately 97.23–98.48% when comparing the control samples to nCeO_2_ NP treated samples.

When considering the lower taxonomic levels “genus”, it was observed that nCeO_2_ NPs could mostly affect the bacterial diversity and abundance of bacterial community as the control samples showed 239 genera whereas treated samples have genera decreasing from 123 to 99 genera. It was also revealed that nCeO_2_ NPs was affecting some bacteria especially unclassified ones while enhancing others and this was revealed when the abundance was higher in treated samples than in the control. The present study revealed the control samples were dominated by unclassified_*Comamonadaceae*, unclassified_*Moraxellaceae*, unclassified_*Pseudomonadaceae*, *Novispirillum*, *Fusibacter*, unclassified_*Enterobacteriaceae*, unclassified_*Xanthomonadaceae*, *Shewanella*, *Proteocatella*, unclassified_*Carnobacteriaceae*, *Acinetobacter*, *Proteiniclasticum* and *Trichococcus* occupying approximately 78.49% of the total community. This was also confirmed while investigating the impact of nCeO_2_-NPs on microbial cell structure using SEM. SEM images revealed that the microbial biomass were damaged and decreased over the increase of nCeO_2_-NPs concentration. Furthermore, samples had more rod-shaped microorganism that can be associated to *Acinetobacter*, *Comamonadaceae*, *Moraxellaceae, Pseudomonadaceae* despite of the presence of cocci-shaped microorganism such as *Trichococcus* (Fig. 5). Although most the dominant genus was unclassified, it was reported that genera and species belonging to *Comamonadaceae* family are considered as functional bacteria as they classified as denitrifiers (Khan et al. 2002; Sadaie et al. 2007). These authors revealed that the species belonging to these genera can be involved into the removal of phosphate in wastewater. Furthermore, previous studies also reported the predominance of several genera and species belonging to *Moraxella*, *Pseudoxanthomonas*, *Comamonadas* in activated sludge (Naili et al. 2015). Khan et al. (2002) also reported that species belong to *comamonadaceae* are primary degrading denitrifiers in activated sludge.

As the concentration of nCeO_2_ NP increased, samples showed a decrease of approximately 28.6% (20,136 reads) to 57.1% (12,084 reads) reads in the samples treated with 10 mg/L-CeO_2_ and 40 mg/L-CeO_2_, respectively. This was also noted with the number of OTUS which appeared to be approximately 27,967 OTUs from the control samples while the sample with highest nCeO_2_ NP revealed a total of 6433 OTUs. However, the relative abundance of two functional bacterial genera (*Trichococcus* and *Acinetobacter*) was found to alternatively dominate treated sample populations whereas most of those from the control samples saw their growth slowing down and inhibited. Vande Walle et al. (2012) disagreed with the findings from control samples by reporting that *Acinetobacter*, *Aeromonas* and *Trichococcus* as the predominant functional bacterial genera within urban sewer infrastructure. According to Lv et al. (2014), *Trichococcus* is among the most abundant genera responsible for denitrifying and aerobic phosphorus removal in the activated sludge. This genus was found to be enhanced in the present study highlighting that nCeO_2_ NPs are beneficial to their growth in the activated sludge and this similarly to *Acinetobacter*. The importance of *Trichococcus* species was further reported by Scheff et al. (1984) who revealed that their presence from bulking sludge. Despite their presence, the inhibition of phosphate removal from the treated samples as compared to nitrate removal could be due to the drastic inhibition of the activities of enzyme catalysing the degradation of polyphosphate such as adenylate kinase (ADK) and polyphosphate kinase (PPK) (Table 2). These enzymes have been reported as responsible in releasing and taking up phosphorus from the activated sludge, respectively (Chen et al. 2012). Furthermore, since unclassified bacteria appeared to be sensitive to nCeO_2_-NPs and this coupled with the inhibition of phosphate removal, it can be hypothesized that these unclassified bacteria were phosphate accumulating organisms (PAOs). It should be mentioned that the inhibition of phosphate removal is of great concern since this pollutant is considered the main responsible of eutrophication (Kamika et al. 2014). The effect of nCeO_2_ NPs was mostly observed with less abundant bacterial species such as sludge bulking bacterial species (*Dechloromonas* and *Thauera*), ammonia-oxidizing bacterial species (*Zoogloea*, *Methyloversatilis*), denitrifying bacterial species (*Thauera*, *Azoarcus*, *Acidovorax*, *Comamonas*, *Pseudomonas*, *Paracoccus*, *Ochrobactrum*, *Hyphomicrobium* and *Nitrospira*), Sulfate-reducing bacterial genera (*Desulfomicrobium* and *Paracoccus*), phosphate removing bacteria genera (*Dechloromonas*, *Azospira*, unclassified_*Burkholderiales*_incertae_sedis), and bacteria involved in flocs stabilization (*Caldilinea*) which showed an significant decrease over the gradual increase of nCeO NPs (Juretschko et al. 2002; Daims et al. 2006). Nevertheless, this did not affect the removal of COD and nitrate from the treated samples. This appeared to be contradictory as the enzymes associated with denitrification were affected by the increase of nCeO_2_ (Fig. 4). However, these enzymes have differently been affected with respect to nCeO_2_ NPs concentration. Nitrite reductase was less sensitive toward nCeO_2_ NPs increase than nitrate reductases. It has been reported that denitrifying bacteria convert nitrate into nitrogen gas via an enzymatic pathway consisting of four successive steps involving nitrate reductase (NaR), nitrite reductase (NiR), nitric oxide reductase, and nitrous oxide reductase in the periplasm and/or cytoplasm (Adav et al. 2010). Although the nCeO_2_ NPs were noted to promote the growth of some bacterial species while slowing those of others, it was unclear to know the real cause of such behavior as unclassified bacteria were mostly affected by the toxic effects of test NPs. Similar to the present study, Das et al. (2012) reported that bacteria community have four general exposure responses namely (1) intolerant, (2) impacted but recovering, (3) tolerant, and (4) stimulated when exposed to nanoparticles such as nAg-NP. Meli et al. (2016) also revealed that moderate concentrations of nanoparticles such as nZnO could accelerate the growth of some types of denitrifying bacteria and promote the growth of some pathogenic bacteria, and can also destroy the integrity of the cell membrane of *Nitrosomonas europaea*. Although, very little information is available on how these nCeO_2_ NPs affect microbial communities in activated sludge, effect of other NPs have been reported. The impact of nCeO_2_ NP on microbial community has also been reported by Antisari et al. (2013) who revealed that though microbial biomass was not statistically affected by nCeO_2_ NPs, the microbial stress or changes was noted. Beside of nCeO_2_, other engineered metal oxides-NPs such as nAg NPs (Das et al. 2012), nZnO NPS (Meli et al. 2016) and TiO2 NPs (Shah et al. 2014) have also been reported to have toxic effects on microbial community from several ecosystem. Jeong et al. (2014) also revealed the impact of nAg-NPs on bacterial community from wastewater treatment systems. These authors revealed that nitrifying bacteria are most susceptible to NPs such as nAg.

In conclusion, the present study provided a comprehensive insight in the effect of nCeO-NPs to bacterial community structure of activated sludge using Illumina sequencing. The present results revealed that *Proteobacteria* was the most predominant phylum in both treated and not-treated samples with nCeO_2_ NPs with exception in the 30 mg-nCeO_2_/L and 40 mg-nCeO_2_/L treated samples. The number of genus in control samples was found to be the lowest compared to treated samples as a large number of orders could not be classified. Despite of inhibiting some bacterial species especially the less abundant and unclassified ones, nCeO_2_ NPs appeared to enhance the growth of some bacterial species such as *Trichococcus* and *Acinetobacter*. Nevertheless, this enhancement did not increase the removal of phosphate in the treated samples. The results can extend our biological knowledge by revealing that nCeO_2_ NPs at moderate concentration could be beneficial as they enhanced some bacterial species involved nitrification, denitrification, and phosphorylation cycles in EBPR. More studies are needed to further understand the mechanism involved in the enhancement of bacteria growth by nCeO_2_ NPs as well as the inhibition of phosphate due to continuous addition of nCeO_2_-NPs.

## Additional file

**Additional file 1: Table S1.** Pairwise bacterial community similarity between reactors using Jaccard index at 3% nucleotide cutoff level. **Table S2.** Relative abundance of bacterial classes in the reactors. **Table S3.** Relative abundance of bacterial orders in the reactors. **Table S4.** Relative abundance of bacterial families in the reactors. **Table S5.** Relative abundance of bacterial genera in the reactors.

## Abbreviations

ADK: adenylate kinase
COD: chemical oxygen demand
DRA: DNA data base of Japan sequence read archive
DO: dissolved oxygen
EC: electrical conductivity
EBPR: enhanced biological phosphorus removal
NaR: nitrate reductase
NiR: nitrite reductase
NPs: nanoparticles
OTUs: operational taxonomic units
PPK: polyphosphate kinase
RDP: ribosomal database project
SEM: scanning electronic microscope
WWTP: wastewater treatment plant

## References

[CR1] Adav SS, Lee DJ, Lai JY (2010). Enhanced biological denitrification of high concentration of nitrite with supplementary carbon source. Appl Microbiol Biotechnol.

[CR2] Antisari LV, Carbone S, Gatti A, Vianello G, Nannipieri P (2013). Toxicity of metal oxide (CeO_2_, Fe_3_O_4_, SnO_2_) engineered nanoparticles on soil microbial biomass and their distribution in soil. Soil Biol Biochem.

[CR605] APHA (2001) Standard methods for the examination of water and wastewater, 20th edn. American Public Health Association, Washington, DC

[CR3] Arnold MC, Badireddy AR, Wiesner MR, Di Giulio RT, Meyer JN (2013). Cerium oxide nanoparticles are more toxic than equimolar bulk cerium oxide in caenorhabditis elegans. Arch Environ Contam Toxicol.

[CR300] Aruoja V, Dubourguier HC, Kasemets K, Kahru A (2009) Toxicity of nanoparticles of CuO, ZnO and TiO(2) to microalgae *Pseudokirchneriella subcapitata*. Sci Total Environ 407:1461–1468 10.1016/j.scitotenv.2008.10.053 19038417

[CR4] Asati A, Santra S, Kaittanis C, Nath S, Perez JM (2009). Oxidase-like activity of polymer-coated cerium oxide nanoparticles. Angew Chem Int Ed.

[CR5] Bouzigues C, Gacoin T, Alexandrou A (2011). Biological applications of rare-earth based nanoparticles. ACS Nano.

[CR200] Bumajdad A, Eastoe J, Mathew A (2009) Cerium oxide nanoparticles prepared in self-assembled systems. Adv Colloid Interface Sci 147–148:56–66 10.1016/j.cis.2008.10.004 19027889

[CR6] Chen JP, Patil S, Seal S, McGinnis JF (2006). Rare earth nanoparticles prevent retinal degeneration induced by intracellular peroxides. Nat Nanotechnol.

[CR7] Chen Y, Chen H, Zheng X, Mu H (2012). The impacts of silver nanoparticles and silver ions on wastewater biological phosphorous removal and the mechanisms. J Hazard Mater.

[CR8] Chen J, Tang YQ, Li Y, Nie Y, Hou L, Li XQ, Wu XL (2014). Impacts of different nanoparticles on functional bacterial community in activated sludge. Chemosphere.

[CR2000] Cole JR, Wang Q, Fish JA, Chai B, McGarrell DM, Sun Y, Brown CT, Porras-Alfaro A, Kuske CR, Tiedje JM (2014) Ribosomal database project: data and tools for high throughput rRNA analysis. Nucl Acids Res 42(Database issue):D633–D642. doi:10.1093/nar/gkt1244 10.1093/nar/gkt1244 PMC3965039 24288368

[CR9] Daims H, Taylor MW, Wagner M (2006). Wastewater treatment: a model system for microbial ecology. Trends Biotechnol.

[CR10] Das P, Williams CJ, Fulthorpe RR, Hoque ME, Metcalfe CD, Xenopoulos MA (2012). Changes in bacterial community structure after exposure to silver nanoparticles in natural waters. Environ Sci Technol.

[CR900] Edgar RC, Haas BJ, Clemente, JC, Quince C, Knight R (2011) UCHIME improves sensitivity and speed of chimera detection. Bioinformatics 27(16):2194–2200. doi:10.1093/bioinformatics/btr381 10.1093/bioinformatics/btr381 PMC3150044 21700674

[CR11] Federico A, Morgillo F, Tuccillo C, Ciardiello F, Loguercio C (2007). Chronic inflammation and oxidative stress in human carcinogenesis. Int J Cancer.

[CR100] Goharshadia EK, Samieea S, Nancarrowc P (2011) Fabrication of cerium oxide nanoparticles: characterization and optical properties. J Colloid Interface Sci 356(2):473–480 10.1016/j.jcis.2011.01.063 21316699

[CR12] Hu Z, Haneklaus S, Sparovek G, Schnug E (2006). Rare earth elements in soils. Commun Soil Sci Plant Anal.

[CR13] Hussain S, Al-Nsour F, Rice AB, Marshburn J, Yingling B, Ji ZX, Zink JI, Walker NJ, Garantziotis S (2012). Cerium dioxide nanoparticles induce apoptosis and autophagy in human peripheral blood monocytes. ACS Nano.

[CR14] Jeong E, Im WT, Kim DH, Kim MS, Shin HS, Chae SR (2014). Different susceptibilities of bacterial community to silver nanoparticles in wastewater treatment systems. J Environ Sci Health A Tox Hazard Subst Environ Eng.

[CR15] Juretschko S, Loy A, Lehner A, Wagner M (2002). The microbial community composition of a nitrifying-denitrifying activated sludge from an industrial sewage treatment plant analyzed by the full-cycle rRNA Approach. Syst Appl Microbiol.

[CR16] Kamika I, Coetzee M, Mamba BB, Msagati T, Momba MNB (2014). The impact of microbial ecology and chemical profile on the enhanced biological phosphorus removal (EBPR) process: a case study of Northern Wastewater Treatment Works, Johannesburg. Int J Environ Res Public Health.

[CR17] Karakoti AS, Tsigkou O, Yue S, Lee PD, Stevens MM, Jones JR, Seal S (2010). Rare earth oxides as nanoadditives in 3-D nanocomposite scaffolds for bone regeneration. J Mater Chem.

[CR700] Kenji A, Riu S, Hiroshi N (1981) Isolation and identification of respiratory nitrate reductase-producing bacteria from soil and production of the enzyme. Agric Biol Chem 45:817–822

[CR18] Khan ST, Horiba Y, Yamamoto M, Hiraishi A (2002). Members of the family *Comamonadaceae* as primary Poly(3-hydroxybutyrate-co-3-hydroxyvalerate)-degrading denitrifiers in activated sludge as revealed by a polyphasic approach. Appl Environ Microbiol.

[CR500] Klindworth A, Pruesse E, Schweer T, Peplies J, Quast C, Horn M, Glöckner FO (2013) Evaluation of general 16S ribosomal RNA gene PCR primers for classical and next-generation sequencing-based diversity studies. Nucleic Acids Res 41(1):e1 10.1093/nar/gks808 PMC3592464 22933715

[CR19] Li M, Shi P, Xu C, Ren JS, Qu XG (2013). Cerium oxide caged metal chelator: anti-aggregation and anti-oxidation integrated H_2_O_2_-responsive controlled drug release for potential Alzheimer’s disease treatment. Chem Sci.

[CR20] Lin WS, Huang YW, Zhou XD, Ma YF (2006). Toxicity of cerium oxide nanoparticles in human lung cancer cells. Int J Toxicol.

[CR21] Liu Y, Zhang T, Fang HHP (2005). Microbial community analysis and performance of a phosphate-removing activated sludge. Bioresour Technol.

[CR603] Lv XM, Shao MF, Li CL, Gao XL, Sun FY (2014) A comparative study of the bacterial community in denitrifying and traditional enhanced biological phosphorus removal processes. Microbes Environ 29(3):261–268 10.1264/jsme2.ME13132 PMC4159037 24964811

[CR22] Mandoli C, Pagliari F, Pagliari S, Forte G, Di Nardo P, Licoccia S, Traversa E (2010). Stem cell aligned growth induced by CeO_2_ nanoparticles in PLGA scaffolds with improved bioactivity for regenerative medicine. Adv Funct Mater.

[CR23] Mariani E, Polidori MC, Cherubini A, Mecocci P (2005). Oxidative stress in brain aging, neurodegenerative and vascular diseases: an overview. J Chromatogr B.

[CR24] Maritim AC, Sanders RA, Watkins JB (2003). Diabetes, oxidative stress, and antioxidants: a review. J Biochem Mol Toxicol.

[CR604] Meli K, Kamika I, Keshri J, Momba MNB (2016) The impact of zinc oxide nanoparticles on the bacterial microbiome of activated sludge systems. Sci Rep 6:39176. doi:10.1038/srep39176 10.1038/srep39176 PMC5155299 27966634

[CR25] Mittal S, Pandey AK (2014). Cerium oxide nanoparticles induced toxicity in human lung cells: role of ROS mediated DNA damage and apoptosis. BioMed Res Int.

[CR400] Monteiro MIC, Ferreira FN, Oliveira NMM, Avila AK (2003) Simplified version of the sodium salicylate method for nitrate analysis in drinking waters. Anal Chim Acta 477(1):125–129

[CR26] Naili O, Benoumis M, Benammar L (2015). Screening of bacteria isolated from activated sludges for phosphate removal from wastewater. J Appl Environ Biol Sci.

[CR602] Oehmen A, Lemos PC, Carvalho G, Yuan Z, Keller J, Blackall LL, Reis MAM (2007) Advances in enhanced biological phosphorus removal: from micro to macro scale. Water Res 41:2271–2300 10.1016/j.watres.2007.02.030 17434562

[CR27] Pelletier DA, Suresh AK, Holton GA, McKeown CK, Wang W, Gu B, Mortensen NP, Allison DP, Joy DC, Allison MR, Brown SD, Phelps TJ, Doktycz MJ (2010). Effects of engineered cerium oxide nanoparticles on bacterial growth and viability. Appl Environ Microbiol.

[CR28] Sadaie T, Sadaie A, Takada M, Hamano K, Ohnishi J, Ohta N, Matsumoto K, Sadaie Y (2007). Reducing sludge production and the domination of *Comamonadaceae* by reducing the oxygen supply in the wastewater treatment procedure of a food-processing factory. Biosci Biotechnol Biochem.

[CR29] Sanz JL, Kochling T (2007). Molecular biology techniques used in wastewater treatment: an overview. Process Biochem.

[CR30] Scheff G, Salcher O, Lingens F (1984). *Trichococcus flocculiformis* gen. nov. sp. nov. A new gram-positive filamentous bacterium isolated from bulking sludge. Appl Microbiol Biotechnol.

[CR800] Schloss PD, Westcott SL, Ryabin T, Hall JR, Hartmann M, Hollister EB, Lesniewski RA, Oakley BB, Parks DH, Robinson CJ, Sahl JW, Stres B, Thallinger GG, van Horn DJ, Weber CF (2009) Introducing mothur: open-source, platform-independent, community-supported software for describing and comparing microbial communities. Appl Environ Microbiol 75(23):7537–7541 10.1128/AEM.01541-09 PMC2786419 19801464

[CR600] Sekar S, Zintchem AAEA, Keshri J, Kamika I, Momba MNB (2014) Bacterial profiling in brine samples of the Emalahleni Water Reclamation Plant, South Africa, using 454-pyrosequencing method. FEMS Microbiol Lett 359:55–63 10.1111/1574-6968.12557 25168269

[CR601] Seviour RJ, Mino T, Onuki M (2003) The microbiology of biological phosphorus removal in activated sludge systems. FEMS Microbiol Rev 27:99–127 10.1016/S0168-6445(03)00021-4 12697344

[CR31] Shah V, Jones J, Dickman J, Greenman S (2014). Response of soil bacterial community to metal nanoparticles in biosolids. J Hazard Mater.

[CR32] Sidat M, Bux F, Kasan HC (1997). Phosphate accumulation by bacteria isolated from activated sludge. Water SA.

[CR33] Srinivas A, Rao PJ, Selvam G, Murthy PB, Reddy PN (2011). Acute inhalation toxicity of cerium oxide nanoparticles in rats. Toxicol Lett.

[CR34] Taylor NS, Merrifield R, Williams TD, Chipman JK, Lead JR, Viant MR (2015). Molecular toxicity of cerium oxide nanoparticles to the freshwater alga *Chlamydomonas reinhardtii* is associated with supra-environmental exposure concentrations. Nanotoxicology.

[CR35] Thomsen TR, Kong Y, Nielsen PH (2007). Ecophysiology of abundant denitrifying bacteria in activated sludge. FEMS Microbiol Ecol.

[CR36] Vande Walle JL, Goetz GW, Huse SM, Andreischcheva EN, Sogin ML, Hoffmann RG, Yan K, McLellan SL (2012). *Acinetobacter*, *Aeromonas*, and *Trichococcus* dominate the microbial community within urban sewer infrastructure. Environ Microbiol.

[CR1000] Wang Q, Garrity GM, Tiedje JM, Cole JR (2007) Naïve Bayesian classifier for rapid assignment of rRNA sequences into the new bacterial taxonomy. Appl Environ Microbiol 73(16):5261–5267 10.1128/AEM.00062-07 PMC1950982 17586664

[CR37] Xu C, Qu X (2014). Cerium oxide nanoparticle: a remarkably versatile rare earth nanomaterial for biological applications. NPG Asia Mater.

[CR38] Xu C, Lin Y, Wang J, Wu L, Wei W, Ren J, Qu X (2013). Nanoceria-triggered synergetic drug release based on CeO_2_-capped mesoporous silica host–guest interactions and switchable enzymatic activity and cellular effects of CeO_2_. Adv Healthc Mater.

[CR39] Zhang T, Shao MF, Ye L (2012). 454 Pyrosequencing reveals bacterial diversity of activated sludge from 14 sewage treatment plants. ISME J.

