# Supplementary material for: Impacts of cerium oxide nanoparticles on bacterial community in activated sludge
Source: AMB Express. 2017 Mar 15;7:63. doi: 10.1186/s13568-017-0365-6 (PMC5352701; doi:10.1186/s13568-017-0365-6)
Supplement: Supplementary file 1 — Additional file 1: Table S1. Pairwise bacterial community similarity between reactors using Jaccard index at 3% nucleotide cutoff level. Table S2. Relative abundance of bacterial classes in the reactors. Table S3. Relative abundance of bacterial orders in the reactors. Table S4. Relative abundance of bacterial families in the reactors. Table S5. Relative abundance of bacterial genera in the reactors. [file 13568_2017_365_MOESM1_ESM.pdf]

Journal Title: AMB Express

Manuscript title: Impacts of cerium oxide nanoparticles on bacterial community in activated sludge

Kamika I[a],

[a]Department of Environmental Sciences, College of Agriculture and Environmental Science, University of South Africa, PO Box 392 UNISA 0003, South Africa

Corresponding author: Kamika I: Tel: +27 11 670 9745; email: alainkamika@gmail.com

Tekere M[a]

[a]Department of Environmental Sciences, College of Agriculture and Environmental Science, University of South Africa, PO Box 392 UNISA 0003, South Africa

Email: kamiki@unisa.ac.za

Table S1: Pairwise bacterial community similarity between reactors using Jaccard index at 3% nucleotide cutoff level

	S_A (10 mg/L)	S_B (20 mg/L)	S_C (30 mg/L)	S_D (40 mg/L)	Control
S_A (10 mg/L)	0	0.50124145	0.4793955	0.54474527	0.999527
S_B (20 mg/L)	0.50124145	0	0.49129128	0.57615817	0.99938214
S_C (30 mg/L)	0.4793955	0.49129128	0	0.53497803	0.9997715
S_D (40 mg/L)	0.54474527	0.57615817	0.53497803	0	0.99970865
Control	0.999527	0.99938214	0.9997715	0.99970865	0

Table S2: Relative abundance of bacterial classes in the reactors

class	Control	S_A (10 mg/L)	S_B (20 mg/L)	S_C (30 mg/L)	S_D (40 mg/L)
Gammaproteobacteria	17198	9383	7622	6319	5300
Betaproteobacteria	756	955	1203	1896	2019
Alphaproteobacteria	1230	228	222	242	117
Epsilonproteobacteria	2	1	0	1	0
Deltaproteobacteria	23	1	1	3	3
Flavobacteriia	45	243	292	285	466
Cytophagia	1	35	3	0	1
Sphingobacteriia	5	42	25	26	10
Bacteroidetes_incertae_sedis	0	1	1	0	0
Bacteroidia	139	0	6	5	1
Bacilli	714	8250	4447	4970	3478
Clostridia	888	29	44	15	26
Erysipelotrichia	1	5	3	1	0
Negativicutes	6	0	3	0	1
Planctomycetia	11	16	11	0	4
Actinobacteria	429	91	102	59	59
Caldilineae	69	12	17	8	4
Anaerolineae	2	8	17	7	8
Chloroflexia	0	0	1	1	1
Nitrospira	2	4	1	1	0
Subdivision3	0	1	1	0	0
Verrucomicrobiae	1	0	1	0	2
Acidobacteria_Gp4	1	1	0	0	0
Acidobacteria_Gp16	0	0	2	0	0
Acidobacteria_Gp6	0	0	0	1	0
Fusobacteriia	2	0	2	1	0
Chlamydiia	1	0	0	0	1
Mollicutes	1	0	0	0	0
Ignavibacteria	1	0	0	0	0
Synergistia	1	0	0	0	0

unclassified_"*Proteobacteria*"	1882	288	208	191	232
unclassified_*Firmicutes*	105	54	25	22	29
unclassified_"*Chloroflexi*"	96	28	38	17	16
unclassified_"*Bacteroidetes*"	55	32	38	10	17
unclassified_"*Actinobacteria*"	9	1	1	0	0
unclassified_"*Acidobacteria*"	1	0	0	0	0

Table S3: Relative abundance of bacterial orders in the reactors

order	Control	S_A (10 mg/L)	S_B (20 mg/L)	S_C (30 mg/L)	S_D (40 mg/L)
Pseudomonadales	2401	7336	5251	5042	3844
Xanthomonadales	291	894	358	864	762
Alteromonadales	345	232	425	25	30
Aeromonadales	52	119	236	15	5
Chromatiales	32	6	8	1	0
Enterobacteriales	469	3	10	5	242
Legionellales	0	3	0	0	0
Burkholderiales	508	828	1142	1848	1977
Rhodocyclales	41	95	38	22	19
Neisseriales	33	2	1	2	0
Sphingomonadales	2	82	83	76	31
Caulobacterales	3	95	42	113	49
Rhizobiales	149	14	21	21	12
Rhodobacterales	342	10	7	14	9
Rhodospirillales	435	6	58	4	6
Campylobacterales	2	1	0	1	0
Desulfovibrionales	20	1	0	1	1
Myxococcales	0	0	1	1	1
Bdellovibrionales	2	0	0	1	1
Flavobacteriales	45	243	292	285	466
Cytophagales	1	35	3	0	1
Sphingobacteriales	5	42	25	26	10
Bacteroidales	139	0	6	5	1
Lactobacillales	432	8030	4336	4876	3368
Bacillales	139	1	2	0	0
Clostridiales	883	29	44	15	26
Erysipelotrichales	1	5	3	1	0
Selenomonadales	6	0	3	0	1
Planctomycetales	11	16	11	0	4
Coriobacteriales	0	3	0	1	1
Actinomycetales	384	70	75	42	41
Bifidobacteriales	0	0	0	2	0
Acidimicrobiales	4	3	12	3	3
Solirubrobacterales	1	0	0	0	0
Caldilineales	69	12	17	8	4

Anaerolineales	2	8	17	7	8
Chloroflexales	0	0	0	1	1
Nitrospirales	2	4	1	1	0
Verrucomicrobiales	1	0	1	0	2
Fusobacteriales	2	0	2	1	0
Chlamydiales	1	0	0	0	1
Acholeplasmatales	1	0	0	0	0
Ignavibacteriales	1	0	0	0	0
Synergistales	1	0	0	0	0
unclassified_*Alphaproteobacteria*	299	21	11	14	10
unclassified_*Gammaproteobacteria*	13608	790	1334	367	417
unclassified_*Betaproteobacteria*	174	30	22	24	23
unclassified_*Deltaproteobacteria*	1	0	0	0	0
unclassified_*Bacilli*	143	219	109	94	110
unclassified_*Clostridia*	5	0	0	0	0
unclassified_*Actinobacteria*	32	15	15	11	14
unclassified_*Chloroflexia*	0	0	1	0	0
unclassified_*Acidobacteria*_Gp4	1	0	0	0	0

Table S4: Relative abundance of bacterial families in the reactors

family	Control	S_A (10 mg/L)	S_B (20 mg/L)	S_C (30 mg/L)	S_D (40 mg/L)
Moraxellaceae	487	7107	5111	4697	3404
Pseudomonadaceae	1826	211	133	320	423
Xanthomonadaceae	289	890	357	861	761
Alteromonadaceae	7	211	27	4	16
Shewanellaceae	147	14	348	18	9
Aeromonadaceae	52	119	236	15	4
Succinivibrionaceae	0	0	0	0	1
Chromatiaceae	32	6	8	1	0
Enterobacteriaceae	469	3	10	5	242
Legionellaceae	0	3	0	0	0
Comamonadaceae	58	742	1019	1757	1783
Burkholderiales_incertae_sedis	31	7	33	7	6
Alcaligenaceae	6	17	12	6	9
Oxalobacteraceae	0	0	1	0	74
Burkholderiaceae	0	0	0	0	1
Rhodocyclaceae	41	95	38	22	19
Neisseriaceae	33	2	1	2	0
Sphingomonadaceae	1	69	68	67	22
Erythrobacteraceae	0	3	1	4	1
Caulobacteraceae	3	93	39	111	48
Bradyrhizobiaceae	3	5	9	2	2
Phyllobacteriaceae	0	1	0	0	0
Beijerinckiaceae	1	2	1	0	2

Brucellaceae	6	1	0	0	4
Hyphomicrobiaceae	10	0	1	2	2
Methylocystaceae	41	0	1	1	0
Rhizobiaceae	0	0	0	2	0
Aurantimonadaceae	3	0	0	0	0
Rhizobiales_incertae_sedis	2	0	0	0	0
Xanthobacteraceae	1	0	0	0	0
Rhodobacteraceae	342	10	7	14	9
Acetobacteraceae	39	4	49	1	1
Rhodospirillaceae	357	2	4	3	5
Campylobacteraceae	2	1	0	1	0
Desulfovibrionaceae	2	1	0	1	1
Desulfomicrobiaceae	5	0	0	0	0
Bacteriovoracaceae	2	0	0	1	1
Flavobacteriaceae	37	237	290	283	433
Cryomorphaceae	5	0	0	0	0
Cytophagaceae	1	35	3	0	1
Sphingobacteriaceae	4	28	18	16	7
Cyclobacteriaceae	0	5	1	5	0
Chitinophagaceae	1	2	1	4	3
Porphyromonadaceae	86	0	5	3	0
Bacteroidaceae	50	0	1	0	0
Prevotellaceae	1	0	0	1	1
Carnobacteriaceae	243	7640	3754	4707	3019
Aerococcaceae	0	242	449	99	265
Enterococcaceae	0	5	22	1	1
Streptococcaceae	3	1	12	1	2
Lactobacillaceae	0	0	0	0	1
Staphylococcaceae	0	1	0	0	0
Planococcaceae	14	0	2	0	0
Peptostreptococcaceae	216	17	23	8	11
Clostridiaceae 1	146	9	7	3	7
Lachnospiraceae	10	0	3	1	3
Eubacteriaceae	10	0	1	0	0
Ruminococcaceae	3	0	1	0	2
Clostridiales_Incertae Sedis XI	11	0	0	0	1
Clostridiales_Incertae Sedis XII	254	0	0	0	0
Clostridiales_Incertae Sedis XIII	4	0	0	0	0
Erysipelotrichaceae	1	5	3	1	0
Veillonellaceae	0	0	3	0	1
Acidaminococcaceae	4	0	0	0	0
Planctomycetaceae	11	16	11	0	4
Coriobacteriaceae	0	3	0	1	1
Intrasporangiaceae	9	13	11	12	4
Microbacteriaceae	2	0	0	2	0
Promicromonosporaceae	1	0	0	0	0

Dietziaceae	9	7	5	2	7
Mycobacteriaceae	0	1	3	3	0
Nocardiaceae	0	0	0	0	1
Corynebacteriaceae	1	0	0	0	0
Sporichthyaceae	0	0	1	0	0
Nakamurellaceae	1	0	0	0	0
Nocardioidaceae	8	2	5	4	3
Propionibacteriaceae	7	1	0	0	0
Actinomycetaceae	3	0	0	1	0
Bifidobacteriaceae	0	0	0	2	0
Acidimicrobiaceae	1	2	7	3	3
Caldilineaceae	69	12	17	8	4
Anaerolineaceae	2	8	17	7	8
Chloroflexaceae	0	0	0	1	1
Nitrospiraceae	2	4	1	1	0
Verrucomicrobiaceae	1	0	1	0	2
Leptotrichiaceae	2	0	1	0	0
Fusobacteriaceae	0	0	1	1	0
Parachlamydiaceae	1	0	0	0	1
Acholeplasmataceae	1	0	0	0	0
Ignavibacteriaceae	1	0	0	0	0
Synergistaceae	1	0	0	0	0
unclassified_*Pseudomonadales*	88	18	7	25	17
unclassified_*Xanthomonadales*	2	4	1	3	1
unclassified_*Alteromonadales*	191	7	50	3	5
unclassified_*Burkholderiales*	413	62	77	78	104
unclassified_*Rhizobiales*	82	5	9	14	2
unclassified_*Sphingomonadales*	1	10	14	5	8
unclassified_*Caulobacterales*	0	2	3	2	1
unclassified_*Rhodospirillales*	39	0	5	0	0
unclassified_*Desulfovibrionales*	13	0	0	0	0
unclassified_"*Sphingobacteriales*"	0	7	5	1	0
unclassified_"*Flavobacteriales*"	3	6	2	2	33
unclassified_"*Bacteroidales*"	2	0	0	1	0
unclassified_*Lactobacillales*	186	142	99	68	80
unclassified_*Bacillales*	125	0	0	0	0
unclassified_*Clostridiales*	229	3	9	3	2
unclassified_*Selenomonadales*	2	0	0	0	0
unclassified_*Solirubrobacterales*	1	0	0	0	0
unclassified_*Actinomycetales*	225	34	44	11	21

Table S5: Relative abundance of bacterial genera in the reactors

genus	Control	S_A (10 mg/L)	S_B (20 mg/L)	S_C (30 mg/L)	S_D (40 mg/L)
Acinetobacter	137	6222	4596	4270	2974

Enhydrobacter	4	0	0	1	0
Alkanindiges	4	0	0	0	0
Pseudomonas	17	62	47	104	82
Azomonas	0	1	0	1	0
Serpens	92	0	0	0	0
Azotobacter	1	0	0	0	0
Pseudoxanthomonas	40	596	237	567	520
Stenotrophomonas	78	105	55	94	55
Thermomonas	1	0	1	0	0
Alishewanella	7	209	27	4	16
Shewanella	147	14	348	18	9
Aeromonas	27	116	223	15	4
Tolumonas	7	0	2	0	0
Succinivibrio	1	0	0	0	1
Rheinheimera	32	6	6	1	0
Klebsiella	1	0	0	1	2
Salmonella	1	0	0	0	4
Trabulsiella	1	0	0	0	20
Legionella	1	3	0	0	0
Comamonas	1	208	341	663	475
Delftia	1	3	17	43	11
Acidovorax	1	11	26	34	13
Diaphorobacter	1	1	3	4	1
Simplicispira	1	1	1	0	0
Caenimonas	1	0	0	1	0
Variovorax	1	0	0	0	1
Pseudacidovorax	1	0	0	0	0
Albidiferax	2	0	0	0	0
Malikia	1	0	0	0	0
Aquabacterium	4	6	27	5	2
Sphaerotilus	1	0	0	1	1
Rubrivivax	2	0	0	0	1
Achromobacter	2	11	11	6	7
Herminiimonas	1	0	1	0	45
Cupriavidus	1	0	0	0	1
Methyloversatilis	2	63	6	4	4
Zoogloea	1	4	3	1	0
Dechloromonas	1	2	1	0	2
Sulfuritalea	2	1	1	3	4
Azospira	1	2	6	0	0
Thauera	3	2	2	1	0
Azonexus	1	1	0	2	0
Rhodocyclus	1	0	1	0	0
Shinella	1	0	0	0	0
Azoarcus	2	0	0	0	0
Uruburuella	14	2	1	2	0

Novosphingobium	1	10	19	0	1
Sphingobium	1	6	7	13	4
Sphingomonas	1	1	0	2	0
Sphingorhabdus	1	0	1	0	0
Sandaracinobacter	1	0	0	0	0
Porphyrobacter	1	2	1	2	0
Erythromicrobium	1	1	0	2	0
Brevundimonas	2	53	30	79	32
Phenylobacterium	1	4	1	0	0
Caulobacter	1	1	0	1	0
Bosea	1	2	7	1	1
Afipia	1	0	0	1	1
Balneimonas	1	0	0	0	0
Mesorhizobium	1	1	0	0	0
Camelimonas	1	0	0	0	1
Ochrobactrum	1	1	0	0	0
Brucella	2	0	0	0	0
Pseudochrobactrum	1	0	0	0	0
Hyphomicrobium	1	0	0	1	1
Devosia	1	0	0	1	0
Prosthecomicrobium	8	0	0	0	1
Pleomorphomonas	41	0	0	1	0
Rhizobium	1	0	0	2	0
Martelella	2	0	0	0	0
Vasilyevaea	2	0	0	0	0
Rhodobacter	1	1	1	2	0
Gemmobacter	38	1	1	4	2
Paracoccus	1	1	1	1	1
Pannonibacter	3	0	0	0	0
Roseomonas	1	2	40	0	0
Acetobacter	1	1	0	0	0
Defluviicoccus	1	1	1	1	1
Telmatospirillum	15	0	1	0	0
Novispirillum	283	0	2	1	2
Insolitispirillum	39	0	0	0	1
Azospirillum	6	0	0	0	1
Rhodocista	1	0	0	0	0
Arcobacter	1	1	0	1	0
Desulfovibrio	1	1	0	0	1
Desulfomicrobium	5	0	0	0	0
Peredibacter	2	0	0	1	1
Cloacibacterium	18	178	228	241	235
Flavobacterium	5	18	34	5	141
Chryseobacterium	1	6	8	17	0
Wautersiella	1	0	1	0	0
Flectobacillus	1	34	2	0	1

Dyadobacter	1	1	0	0	0
Emticicia	1	0	0	0	0
Sphingobacterium	3	5	5	12	4
Pedobacter	1	3	2	0	0
Arcticibacter	1	0	0	0	2
Algoriphagus	1	3	1	1	0
Terrimonas	1	0	0	0	0
Ohtaekwangia	1	1	1	0	0
Parabacteroides	1	0	1	1	0
Dysgonomonas	3	0	4	0	0
Paludibacter	3	0	0	0	0
Bacteroides	11	0	1	0	0
Anaerorhabdus	39	0	0	0	0
Prevotella	1	0	0	0	0
Trichococcus	110	7371	3624	4585	2879
Isobaculum	1	0	0	0	0
Aerococcus	1	239	447	99	262
Enterococcus	1	0	11	1	0
Streptococcus	1	1	11	1	2
Lactococcus	1	0	0	0	0
Lactobacillus	1	0	0	0	1
Staphylococcus	1	1	0	0	0
Planococcus	1	0	1	0	0
Chryseomicrobium	2	0	0	0	0
Clostridium XI	1	10	10	5	4
Proteocatella	141	1	0	0	1
Acetoanaerobium	48	0	0	0	1
Sarcina	1	1	1	0	0
Clostridium sensu stricto	1	4	3	2	2
Anaerobacter	1	1	0	0	1
Proteiniclasticum	133	0	0	0	0
Blautia	1	0	1	1	1
Dorea	1	0	1	0	0
Clostridium XlVb	6	0	1	0	0
Acetobacterium	7	0	0	0	0
Faecalibacterium	1	0	1	0	1
Ruminococcus	1	0	0	0	1
Anaerofilum	2	0	0	0	0
Sedimentibacter	11	0	0	0	1
Fusibacter	235	0	0	0	0
Anaerovorax	4	0	0	0	0
Turicibacter	1	5	3	1	0
Mitsuokella	1	0	1	0	0
Veillonella	1	0	0	0	1
Planctomyces	1	3	2	0	0
Aquisphaera	1	1	0	0	0

Pirellula	1	0	0	0	1
Singulisphaera	1	0	0	0	0
Olsenella	1	1	0	0	0
Collinsella	1	2	0	1	1
Ornithinibacter	1	1	1	0	0
Tetrasphaera	1	0	2	4	0
Okibacterium	1	0	0	0	0
Cellulosimicrobium	1	0	0	0	0
Dietzia	9	7	5	2	7
Mycobacterium	1	1	3	3	0
Gordonia	1	0	0	0	1
Corynebacterium	1	0	0	0	0
Sporichthya	1	0	1	0	0
Nakamurella	1	0	0	0	0
Marmoricola	1	1	0	1	0
Nocardioides	1	0	1	1	1
Pimelobacter	1	0	1	0	0
Tessaracoccus	2	0	0	0	0
Actinobaculum	1	0	0	1	0
Actinomyces	2	0	0	0	0
Bifidobacterium	1	0	0	1	0
Ilumatobacter	1	2	7	2	3
Caldilinea	1	6	8	4	3
Litorilinea	20	0	0	0	0
Longilinea	1	0	1	0	0
Nitrospira	2	4	1	1	0
Subdivision3_genera_incertae_sedis	1	1	1	0	0
Luteolibacter	1	0	1	0	0
Gp4	1	1	0	0	0
Gp16	1	0	2	0	0
Gp6	1	0	0	1	0
Sebaldella	1	0	1	0	0
Saccharibacteria_genera_incertae_sedis	1	0	8	3	6
Acholeplasma	1	0	0	0	0
Ignavibacterium	1	0	0	0	0
unclassified_*Moraxellaceae*	430	342	885	515	426
unclassified_*Pseudomonadaceae*	341	1716	148	86	215
unclassified_*Xanthomonadaceae*	186	171	189	64	200
unclassified_*Alteromonadaceae*	1	0	2	0	0
unclassified_*Aeromonadaceae*	1	18	3	11	0
unclassified_*Chromatiaceae*	1	0	0	2	0
unclassified_*Enterobacteriaceae*	216	469	3	10	4
unclassified_*Comamonadaceae*	1282	54	518	631	1012
unclassified_*Alcaligenaceae*	2	4	6	1	0
unclassified_*Burkholderiales*_incertae_sedis	2	25	1	6	1
unclassified_*Oxalobacteraceae*	29	0	0	0	0

unclassified_*Rhodocyclaceae*	9	31	20	18	11
unclassified_*Neisseriaceae*	1	19	0	0	0
unclassified_*Sphingomonadaceae*	17	0	52	41	52
unclassified_*Erythrobacteraceae*	1	0	0	0	0
unclassified_*Caulobacteraceae*	16	0	35	8	31
unclassified_*Bradyrhizobiaceae*	1	1	3	2	0
unclassified_*Beijerinckiaceae*	1	1	2	1	0
unclassified_*Hyphomicrobiaceae*	1	2	0	1	0
unclassified_*Methylocystaceae*	1	0	0	1	0
unclassified_*Brucellaceae*	4	3	0	0	0
unclassified_"*Aurantimonadaceae*"	1	1	0	0	0
unclassified_*Xanthobacteraceae*	1	1	0	0	0
unclassified_*Rhodobacteraceae*	6	301	7	4	7
unclassified_*Acetobacteraceae*	1	39	1	9	1
unclassified_*Rhodospirillaceae*	1	12	1	0	1
unclassified_*Campylobacteraceae*	1	1	0	0	0
unclassified_*Desulfovibrionaceae*	1	2	0	0	1
unclassified_*Flavobacteriaceae*	57	13	35	19	20
unclassified_*Cryomorphaceae*	1	5	0	0	0
unclassified_*Cytophagaceae*	1	0	0	1	0
unclassified_*Sphingobacteriaceae*	1	1	20	11	4
unclassified_*Chitinophagaceae*	3	0	2	1	4
unclassified_"*Cyclobacteriaceae*"	1	0	2	0	4
unclassified_"*Prevotellaceae*"	1	0	0	0	1
unclassified_"*Porphyromonadaceae*"	1	80	0	0	2
unclassified_*Carnobacteriaceae*	140	132	269	130	122
unclassified_*Enterococcaceae*	1	0	5	11	0
unclassified_*Aerococcaceae*	3	0	3	2	0
unclassified_*Streptococcaceae*	1	2	0	1	0
unclassified_*Planococcaceae*	1	12	0	1	0
unclassified_*Peptostreptococcaceae*	5	27	6	13	3
unclassified_*Clostridiaceae* 1	4	11	3	3	1
unclassified_*Eubacteriaceae*	1	3	0	1	0
unclassified_*Lachnospiraceae*	2	4	0	0	0
unclassified_*Clostridiales*_Incertae Sedis XII	1	19	0	0	0
unclassified_*Ruminococcaceae*	1	1	0	0	0
unclassified_*Veillonellaceae*	1	0	0	2	0
unclassified_*Acidaminococcaceae*	1	4	0	0	0
unclassified_*Planctomycetaceae*	3	10	12	9	0
unclassified_*Intrasporangiaceae*	4	9	12	8	8
unclassified_*Microbacteriaceae*	1	1	0	0	2
unclassified_*Nocardioidaceae*	2	7	1	3	2
unclassified_*Propionibacteriaceae*	1	5	1	0	0
unclassified_*Actinomycetaceae*	1	1	0	0	0
unclassified_*Bifidobacteriaceae*	1	0	0	0	1
unclassified_*Acidimicrobiaceae*	1	0	0	0	1

unclassified_*Caldilineaceae*	1	48	6	9	4
unclassified_*Anaerolineaceae*	8	1	8	16	7
unclassified_*Chloroflexaceae*	1	0	0	0	1
unclassified_*Verrucomicrobiaceae*	2	1	0	0	0
unclassified_"*Fusobacteriaceae*"	1	0	0	1	1
unclassified_"*Leptotrichiaceae*"	1	1	0	0	0
unclassified_*Parachlamydiaceae*	1	1	0	0	0
unclassified_*Synergistaceae*	1	1	0	0	0
